# Comparative transcriptomics and eQTL mapping of response to *Melampsora americana* in selected *Salix purpurea* F_2_ progeny

**DOI:** 10.1186/s12864-021-08254-1

**Published:** 2022-01-22

**Authors:** Dustin G. Wilkerson, Chase R. Crowell, Craig H. Carlson, Patrick W. McMullen, Christine D. Smart, Lawrence B. Smart

**Affiliations:** 1 Horticulture Section, School of Integrative Plant Science, Cornell AgriTech, Cornell University, Geneva, NY 14456 USA; 2 Plant Pathology & Plant-Microbe Biology Section, School of Integrative Plant Science, Cornell AgriTech, Cornell University, Geneva, NY 14456 USA

**Keywords:** *Salix purpurea*, *Melampsora americana*, Shrub willow, Leaf rust, Transcriptome, 3′ RNA-seq, WGCNA, Differential expression, eQTL

## Abstract

**Background:**

*Melampsora* spp. rusts are the greatest pathogen threat to shrub willow (*Salix* spp.) bioenergy crops. Genetic resistance is key to limit the effects of these foliar diseases on host response and biomass yield, however, the genetic basis of host resistance has not been characterized. The addition of new genomic resources for *Salix* provides greater power to investigate the interaction between *S. purpurea* and *M. americana*, species commonly found in the Northeast US. Here, we utilize 3′ RNA-seq to investigate host-pathogen interactions following controlled inoculations of *M. americana* on resistant and susceptible F_2_ *S. purpurea* genotypes identified in a recent QTL mapping study. Differential gene expression, network analysis, and eQTL mapping were used to contrast the response to inoculation and to identify associated candidate genes.

**Results:**

Controlled inoculation in a replicated greenhouse study identified 19 and 105 differentially expressed genes between resistant and susceptible genotypes at 42 and 66 HPI, respectively. Defense response gene networks were activated in both resistant and susceptible genotypes and enriched for many of the same defense response genes, yet the hub genes of these common response modules showed greater mean expression among the resistant plants. Further, eight and six eQTL hotspots were identified at 42 and 66 HPI, respectively. The combined results of three analyses highlight 124 candidate genes in the host for further analysis while analysis of pathogen RNA showed differential expression of 22 genes, two of which are candidate pathogen effectors.

**Conclusions:**

We identified two differentially expressed *M. americana* transcripts and 124 *S. purpurea* genes that are good candidates for future studies to confirm their role in conferring resistance.

**Supplementary Information:**

The online version contains supplementary material available at 10.1186/s12864-021-08254-1.

## Background

Shrub willow (*Salix* spp.) are fast-growing perennials that can be grown as a sustainable source of bioenergy, in riparian buffers, or as ornamentals [1]. *Salix* is incredibly diverse, comprised of over 350 species, with a native range that primarily spans the northern hemisphere, but is cultivated around the world [2]. Of the species found in the northeastern US, naturalized *S. purpurea* has been the focus of bioenergy breeding programs for its high yield, vertical growth habit, and broad resistance to pests and pathogens [3–5]. Genomic resources have been developed for the establishment of *S. purpurea* as a model bioenergy crop, which includes high-quality, annotated reference genomes [6] (https://phytozome-next.jgi.doe.gov). In addition, genetic resources have been generated to better understand the inheritance of key traits used in breeding and selection.

The plant pathogen that is the greatest threat to shrub willow grown in commercial production is willow leaf rust (*Melampsora* spp.) [7–9]. *Melampsora* rusts infecting willow are lesser-known members of the order Pucciniales that includes wheat stem rust (*Puccinia graminis*), coffee rust (*Hemileia vastatrix*) and over 7000 other species [9, 10]. Previous work has identified *M. americana* as the primary contributor to disease epidemics on *S. purpurea* in the northeastern US [11, 12]. Defined as a macrocyclic and heteroecious obligate biotroph, *M. americana* requires an alternate host to complete all five spore stages in its life cycle and cannot be cultured outside of its living host [13, 14]. Aeciospores produced on *Abies balsamea* are the primary source of inoculum, traveling to susceptible willow hosts via winds in the late spring and early summer months [11]. The production and spread of asexual uredospores on willow facilitates rapid host disease development and subsequent significant yield losses [15]. Given the prolific nature of this disease, durable genetic resistance is essential to achieving sustained shrub willow biomass yield. Recent investigations have identified morphological characteristics that may impact rust infection, including stomatal and trichome density [12]. However, the genetic basis for *M. americana* rust resistance in willow is not well understood.

In closely related pathosystems, including poplar rust caused by *M. larici-populina* and flax rust caused by *M. lini*, research has identified quantitative and qualitative rust resistance using candidate gene analysis and quantitative trait loci (QTL) mapping approaches [16–21]. Most research in the *Salix* – *Melampsora* pathosystem has focused on *S. viminalis* and *M. larici-epitea* [22–24]. While *S. viminalis* is well-adapted and popular in European bioenergy willow breeding programs, *S. purpurea* is the most commonly used species in the US. Carlson et al., 2019 [25] identified QTL on chromosomes (chr) 1, 5, and 10 associated with leaf rust resistance in a *S. purpurea* F_2_ population. Hanley et al., 2011 [26] also described a rust resistance QTL, *Salix Rust Resistance 1* (SRR1), on chr 1. Although genetic mapping studies have identified major effect loci involved in rust resistance, specific genes responsible for host resistance in these populations were not characterized.

RNA-seq has been used to demonstrate that differentially expressed genes coincide with the resistance response in many pathosystems, including potato - *Phytophthora infestans* [27], soybean - *Xanthomonas axonopodis* [28], and Verticillium wilt in cotton [29]. In willow, network analysis of 3′ RNA-seq from resistant *S. purpurea* and susceptible *S. viminalis* parents and their segregating F_1_ progeny identified key regulatory hub genes involved in the defense response to potato leafhopper (*Empoasca fabea*) [30]. These hub genes are the most connected genes within a co-expression module that are predicted to be highly influential in regulating the expression of the other genes within their module. Applying expression QTL (eQTL) analysis in a segregating pedigree enables the identification of local *cis* and remote *trans* factors in the genome that regulate the expression levels of key genes correlated with traits of interest. For instance, Mähler et al., 2020 [31] used eQTL analysis to identify a key set of candidate genes that determine leaf shape characteristics in *Populus*.

While much has been learned about willow leaf rust over the past decades [11, 32, 33], no study has specifically investigated the transcriptomes of *M. americana* and *S. purpurea* shortly after inoculation. This project uses 3′ RNA-seq to investigate the post-inoculation expression profiles in resistant and susceptible progeny in a *S. purpurea* F_2_ mapping population [25], as well as in the pathogen, *M. americana*.

## Results

### Preliminary study of differential expression

We conducted a preliminary RNA-seq study by inoculating *M. americana* on reference *Salix* genotypes to determine the optimum time post-inoculation to observe differential expression. We inoculated *S. purpurea* hosts ‘Fish Creek’ and 94006 with uredospores of *M. americana* isolate R15–033-03 and then extracted RNA at 0, 18, 42, 66, 90, and 114 h post inoculation (HPI) from inoculated leaves and un-inoculated control leaves. ‘Fish Creek’ and 94006 were selected as hosts because they are the male parent and female grandparent of the F_2_ mapping population known to be segregating for resistance to *M. americana* [25]. A direct contrast between the inoculated and control treatment for each genotype-by-time was performed to generate a total number of differentially expressed genes (DEGs) up-regulated and down-regulated for each host genotype (Fig. S1).

The total number of DEGs (*p* ≤ 0.05) for ‘Fish Creek’ were 0 (0 HPI), 0 (24 HPI), 5589 (48 HPI), 562 (72 HPI), 1637 (96 HPI), and 3061 (120 HPI), whereas DEGs for 94006 were 0, 0, 3796, 948, 597, and 1293 for each ascending time-point (Additional Fig. 1). Neither parent displayed symptoms of infection during the experiment, however, signs of rust were visibly detected at 210 HPI. While uredospore sporulation appeared greater on ‘Fish Creek’ by 258 HPI, both genotypes were susceptible to the pathogen. The greatest number of DEGs was observed in both genotypes at 48 HPI (Additional Fig. 1). Thus, time points 42 and 66 HPI were selected for the full experiment to capture the maximum host and pathogen response after inoculation.

### Greenhouse inoculation of selected resistant and susceptible F_2_ genotypes

**Fig. 1:**
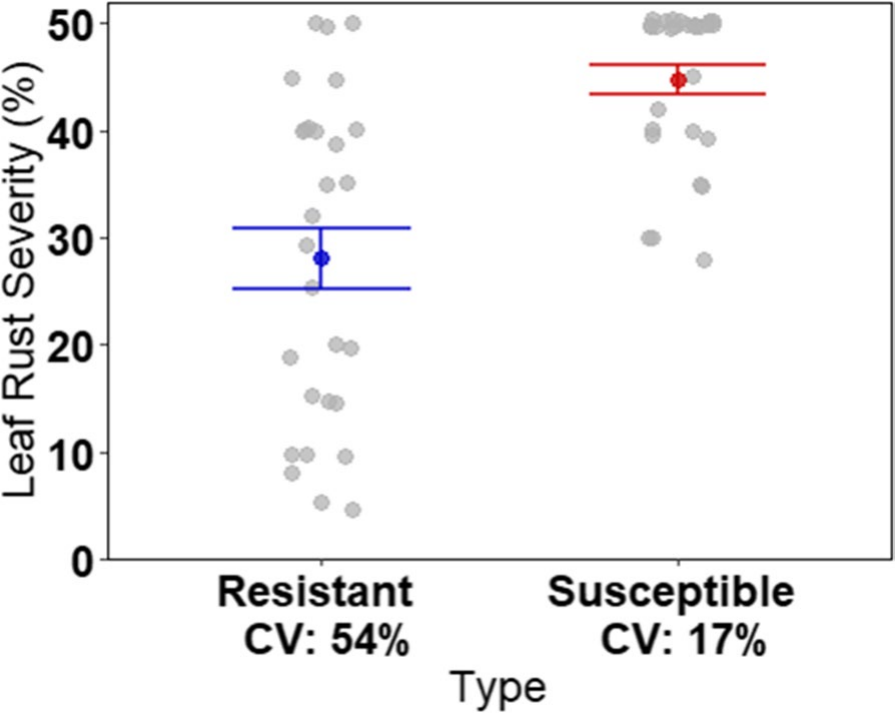
Greenhouse leaf rust severity (%) collected 9 days post inoculation for the resistant and susceptible groups of willow genotypes. Each grey point represents an individual genotype severity while the blue and red points are the mean severity for the resistant and susceptible groups, respectively. Error bars are the standard error of the mean. (CV – Coefficient of Variation)

Based on ratings of rust severity conducted in 2015 and 2017 within a replicated field trial of the *S. purpurea* F_2_ population [25], 28 resistant and 28 susceptible genotypes were selected for controlled inoculation and 3′ RNA-seq alongside the F_2_’s parents and grandparents. At 42 and 66 HPI, leaf discs were collected from two leaves per time point in both the inoculated and control treatments. This experiment was conducted twice in separate greenhouses. Leaf rust severity was assessed in the inoculated treatment at 9 DPI as total percent leaf area coverage of uredospore pustules. The greenhouse ratings were moderately correlated with the 2015 and 2017 field ratings, with Pearson’s correlation values of 0.48 (*p*-value = 9.4 × 10^− 5^) and 0.53 (p-value = 1.6 × 10^− 5^), respectively. The susceptible genotypes had a significantly greater mean rust severity (44.8% - CV: 17%) than the resistant genotypes (28.1% - CV: 54%) based on a t-test (CI = 95%) despite considerably more variability among the resistant genotypes (Fig. 1).

### Differential expression analysis of *S. purpurea* transcripts

**Fig. 2:**
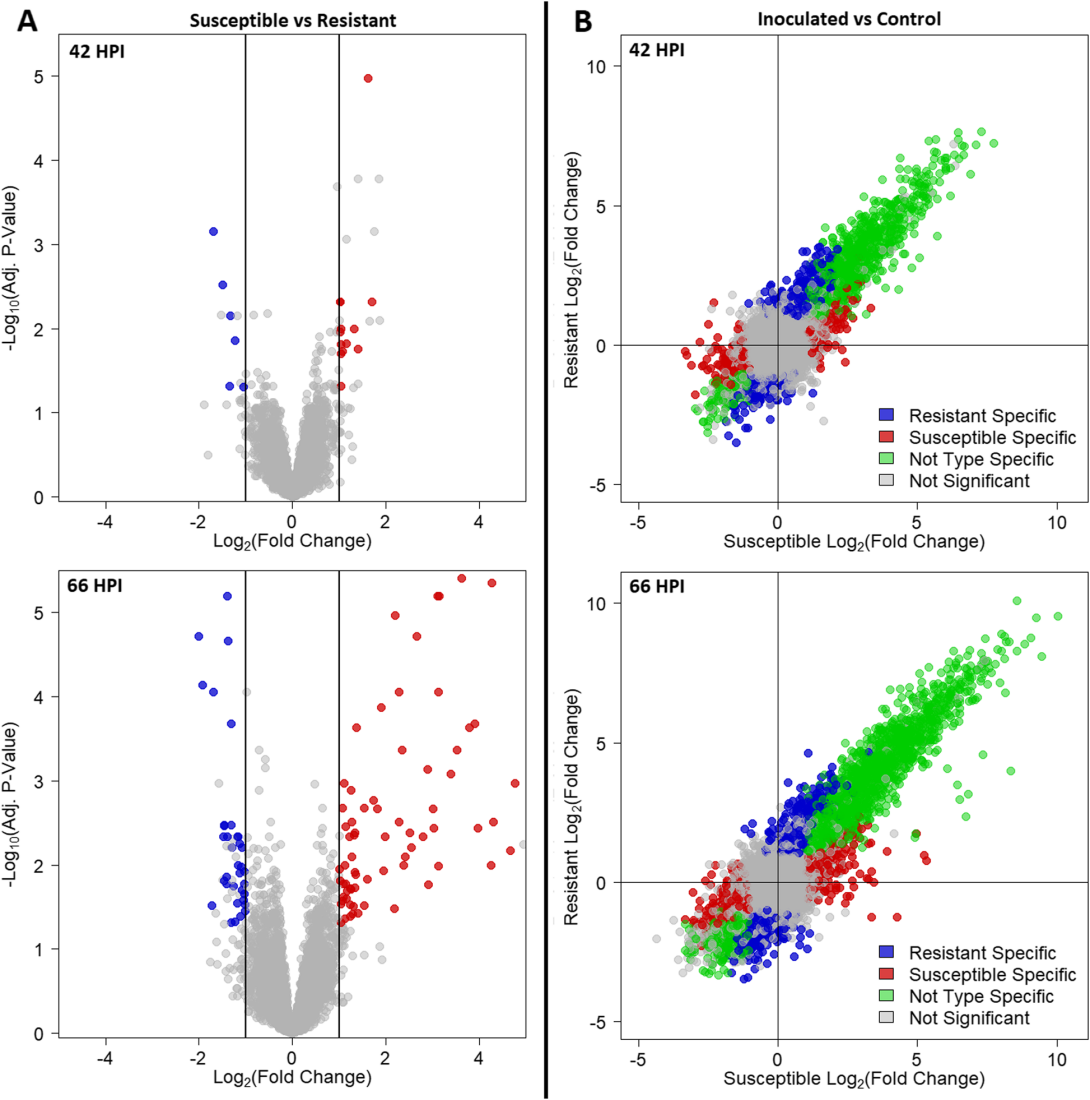
**A** Volcano plots depicting differential expression analysis between inoculated resistant and susceptible groups. Each point represents a gene. Positive Log_2_ Fold Change (LFC) indicates upregulation in the susceptible genotypes (red points) while negative LFC are up-regulated in the resistant genotypes (blue points). **B** Differential expression in inoculated treatments compared with controls plotted as the LFC in expression of the susceptible genotypes on the x axis versus the LFC in expression of the resistant genotypes on the y axis. (HPI – Hours Post Inoculation)

Two separate contrasts in DESeq2 were used to identify differentially expressed genes in this study. In the direct contrast between inoculated susceptible and resistant groups, there were 19 and 105 differentially expressed genes at time points 42 HPI and 66 HPI, respectively (Fig. 2A). Of the 19 DEGs at 42 HPI, six were up-regulated in the resistant genotypes, including a polyubiquitin protein (*UBQ10*), a plasma membrane intrinsic protein (*PIP2;8*), a phosphoglycerate kinase 1 (*PGK1*), a chaperone DnaJ-domain superfamily protein, and two genes of unknown function (DUF). The remaining 13 differentially expressed genes at 42 HPI were up-regulated in the susceptible genotypes and included several genes associated with the flavanone synthesis pathway. The 105 DEGs at 66 HPI consisted of 35 genes up-regulated in the resistant group, while the remaining 70 were up-regulated in the susceptible group. Genes up-regulated at 66 HPI in the resistant group include several involved in defense response such as: wall-associated kinase 2 (*WAK2*), WRKY DNA-binding protein 51, CAP superfamily protein, cytochrome P450, and chitinase A, but as a group, were not significantly enriched for any GO terms. Gene enrichment of the up-regulated susceptible genes were response to heat, stress, and reactive oxygen species (Additional Table 1).

The contrast of inoculated treatments versus uninoculated controls highlighted the response to infection. By performing separate paired analyses for both the resistant and susceptible groups, then intersecting DEGs, variable responses to inoculation were identified at each time-point. We classified DEGs as susceptible-specific, resistant-specific, and not-type-specific (common response between the resistant and susceptible groups). At both time points, the largest group of DEGs was the not-type-specific, positive log_2_-fold change (LFC) group, with 990 and 1862 genes at 42 HPI and 66 HPI respectively (Fig. 2B). All groups of DEGs that were up-regulated after inoculation were enriched for defense response at 42 HPI. However, only the resistant-specific and not-type-specific groups retained enrichment of upregulated defense response genes at 66 HPI. At 66 HPI, the susceptible-specific group lacked genes associated with defense response, but instead displayed upregulation of heat response genes (Additional Table 2). The resistant-specific and the susceptible-specific groups that were down-regulated at 42 HPI were both enriched for chloroplast components, with the susceptible-specific category also enriched for down-regulated ‘response to heat’ genes. There was no significant GO term enrichment at 66 HPI for genes down-regulated in the susceptible-specific category, while both the down-regulated resistant-specific and not-type-specific categories were enriched for genes associated with photosynthesis (Additional Table 2).

### Network analysis of *S. purpurea* transcripts

A comparison between transcriptome-wide expression in the inoculated resistant and susceptible groups was performed in WGCNA, which defined co-expression modules based on correlated gene expression. Each module was randomly assigned a color name by the R package and is only relevant in distinguishing modules within networks, not in making comparisons between them. In this study, modules are referred to either as ‘R-module’ or ‘S-module’ to distinguish between those associated with resistant (R-) or susceptible (S-) plant networks. After removal of outlier samples and genes with low counts, the resistant network retained 75 samples and 16,410 genes, while the susceptible network retained 73 samples and 16,427 genes.

Of the 16,410 genes expressed in the resistant network, 10,176 genes were assigned to 14 modules, while the other 6234 genes were assigned to the ‘grey’ module (unassigned genes). Modules sizes ranged from 33 to 5085 genes, of which nine modules were correlated with time-point (Additional Fig. 2). The largest module ‘R - turquoise’ (*n* = 5085) was positively correlated with time point (r = 0.92) and was the only module enriched for defense-related GO terms in the resistant network (Additional Table 3). The ‘R-blue’ module (*n* = 1853) was inversely correlated with time point (r = − 0.89) and enriched for photosynthesis-related GO terms. A total of 10,977 genes in the susceptible network were placed into 15 modules, with the remaining 5450 placed within the ‘grey’ module. Co-expression modules ranged in size from 25 to 4661 genes, of which 12 were correlated with time point (Additional Fig. 2).

**Fig. 3:**
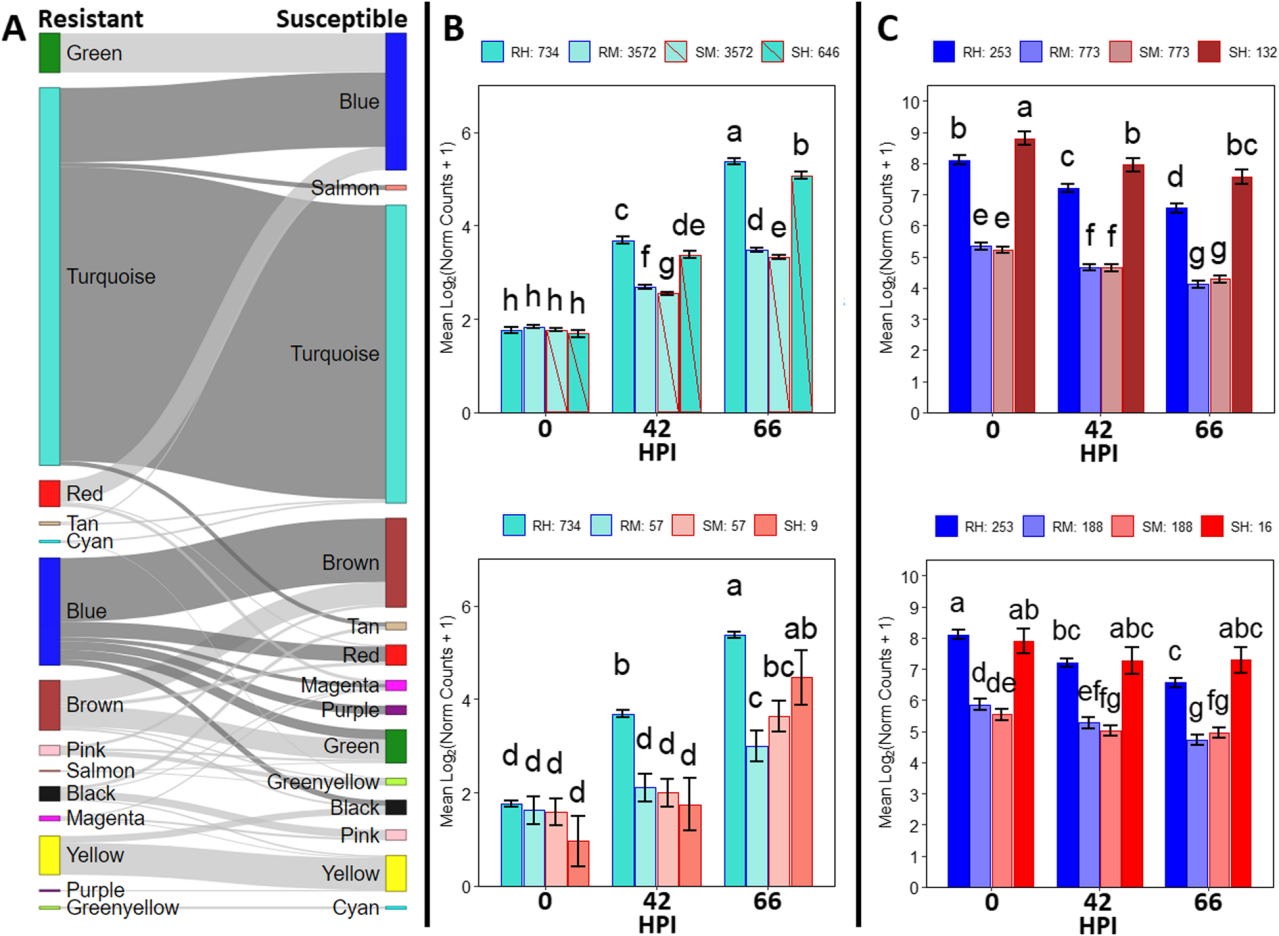
Comparison between the gene expression networks in inoculated resistant and susceptible groups of willow genotypes. **A** Sankey plot of the modules from the resistant network on the left and the susceptible network on the right. Colors represent modules of co-expressed genes. Each connection is significant at the 0.05 level. **B** and **C** Numbers of genes in each group are indicated in the legends above each graph. (RH – Resistant Hub Gene; RM – Resistant Module Gene; SM – Susceptible Module Gene; SH – Susceptible Hub Gene; HPI – Hours Post Inoculation). **B** Mean expression, standard errors, and Fisher’s least significant difference (LSD) group for the ‘R-turquoise’ module compared to the ‘S-turquoise’ and ‘S-salmon’ modules. **C** Mean expression, standard errors, and Fisher’s LSD group for the ‘R-blue’ modules compared to the ‘S-brown’ and ‘S-red’ modules

A hypergeometric test (*p* ≤ 0.05) facilitated a direct comparison between the resistant and susceptible networks to identify significant representation of the susceptible network modules within the ‘R-turquoise’ and ‘R-blue’ resistant modules. The ‘R-turquoise’ and ‘R-blue’ modules shared significant portions of four and six modules, respectively (Fig. 3A). Two modules correlated with time point in the susceptible network with significant ‘R-turquoise’ module representation were ‘S-turquoise’ (*n* = 4661, r = 0.88) and ‘S-salmon’ (*n* = 89, r = 0.51), and were the only susceptible modules enriched for defense-related GO terms (Additional Table 4). Concomitantly, among the six susceptible modules represented within the ‘R-blue’ module and correlated with time point, only the ‘S-brown’ (*n* = 1258, r = − 0.83) and ‘S-red’ (*n* = 264, r = − 0.57) modules were enriched for photosynthetic genes.

To gain insight in the role of hub genes in module composition, hub gene analysis was performed on the ‘R-turquoise’ and ‘R-blue’ modules, in addition to the ‘S-turquoise’, ‘S-salmon’, ‘S-brown’, and ‘S-red’ modules from the susceptible network [34, 35]. Significant differences in mean expression of each module’s hub genes and genes commonly co-expressed across networks were determined using Fisher’s least significant difference (*p* < 0.05). The ‘R-turquoise’ and ‘S-turquoise’ modules had 3572 genes in common, yet at 42 HPI and 66 HPI the mean expression of these genes was greater among resistant genotypes (Fig. 3B). This trend persisted at 42 and 66 HPI among their respective hub genes, whose expression exceeded that of the shared genes. There were only 57 genes shared between the ‘R-turquoise’ and ‘S-salmon’ modules and were not differentially expressed throughout the experiment. However, the expression of ‘S-salmon’ hub genes did not significantly increase until 66 HPI, while ‘R-turquoise’ hub gene expression increased over time.

The ‘R-blue’ module from the resistant network was enriched for photosynthesis-related GO terms and shared commonly co-expressed genes with the ‘S-brown’ and ‘S-red’ modules from the susceptible network that were similarly enriched for photosynthesis (Fig. 3C). There were 773 shared genes between the ‘R-blue’ and ‘S-brown’ modules with similar patterns of decreased expression over time. However, the mean expression of corresponding ‘R-blue’ hub genes was lower at each time point. The genes commonly co-expressed in ‘R-blue’ and ‘S-red’ only accounted for 188 genes that gradually decreased expression through time. Their hub genes, however, show that while the ‘R-blue’ genes decreased after 0 HPI and were beginning to level off by 42 HPI, the ‘S-red’ genes held similar expression throughout.

### eQTL analysis of *S. purpurea* transcripts

**Fig. 4:**
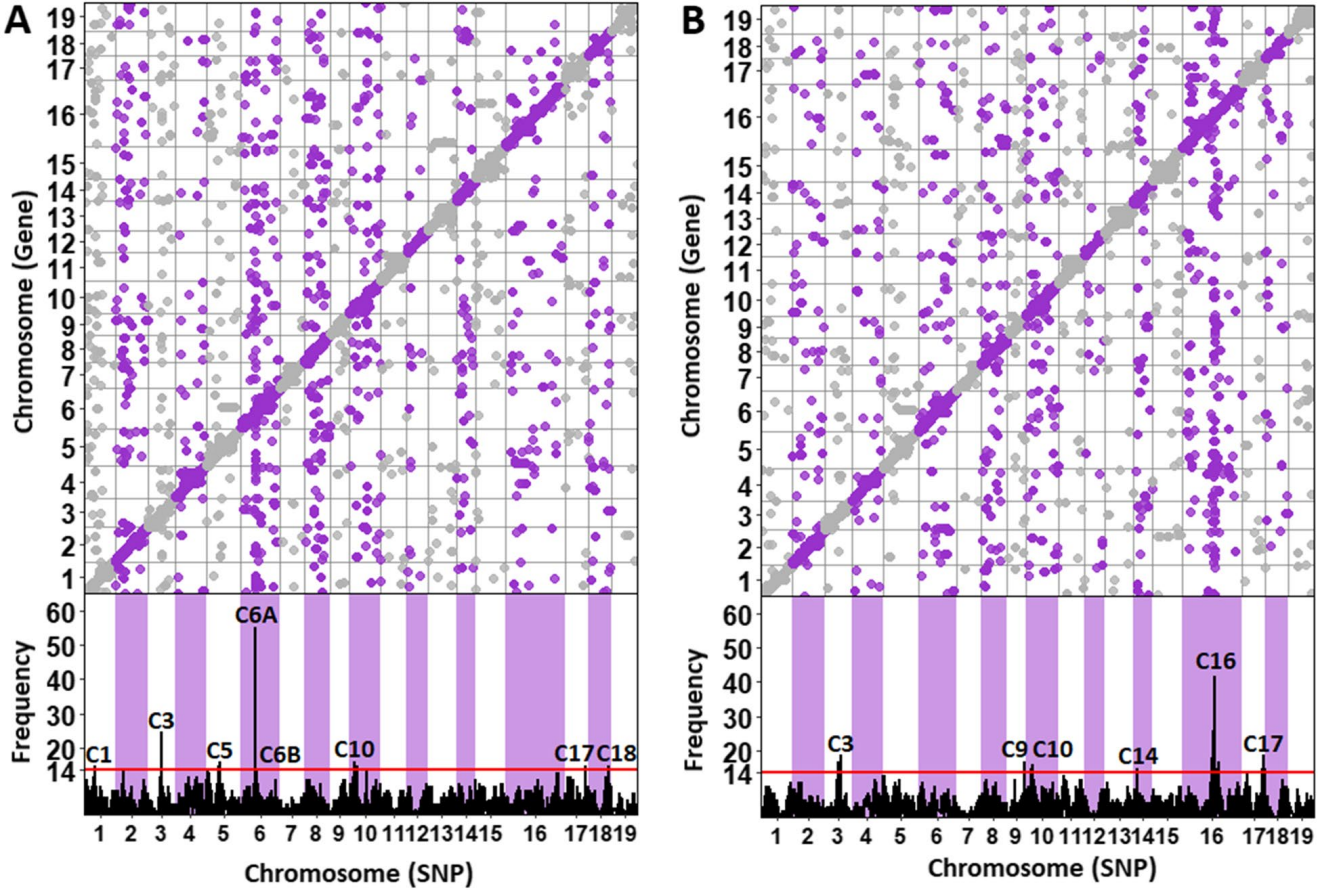
eQTL mapping by time points. **A** 42 h post inoculation (HPI) **B** 66 HPI. For both time points, SNPs are sorted by chromosome across the x-axis. The y-axis of the top panels represents genes mapped to chromosomes. The y-axis of the bottom panels indicates eQTL frequency. The red line indicates the threshold for hotspots, set at 14 eQTL

Mapping of eQTL was performed using 22,068 SNPs and 16,270 genes to interrogate eQTL associated with the response to inoculation, removing those that were detected either at T0 or within the control treatment at the same time point. A total of 38,480 *cis* and 9460 *trans* eQTL were identified at 42 HPI, 45,148 *cis* and 10,638 *trans* eQTL at 66 HPI, and 13,860 *cis* and 1839 *trans* eQTL at both time points (Fig. 4A). Any SNP with more than 14 eQTL, the 95% confidence threshold identified through permutation, was identified as an eQTL hotspot. A hotspot is considered to be a locus influencing the regulation of multiple genes related to allelic phase. Simple correlation analysis (*p* < 0.05) condensed the significant eSNPs into eight eQTL hotspots at 42 HPI and six at 66 HPI (Fig. 4B). Hotspot sizes ranged from 14 to 55 eQTL associations and only three hotspots were enriched for any GO terms (Additional Table 5). The chr 3 hotspot at 42 HPI (C3) was enriched for cell communication and signaling while the chr 6 hotspot at 42 HPI (C6A) was enriched for chloroplast components. The only hotspot at 66 HPI showing GO enrichment was located on chr 16 for photosynthesis and chloroplast components.

### Candidate genes for *S. purpurea* resistance to *M. americana*

Candidate genes which potentially determine a compatible interaction (successful infection) between *S. purpurea* and *M. americana* were identified using the intersection of network analysis, differential expression, and eQTL mapping. Candidate genes were defined as the hub genes of modules found to be enriched for plant defense-related terms and differentially expressed either between resistant and susceptible genotypes or between the inoculated and control treatments. While associations with an eQTL hotspot for response to inoculation were not required for identification as candidate genes, it does aid in prioritization for further research. We identified candidate genes associated with the defense response enriched ‘R-turquoise’ module at 42 HPI (*n* = 31) and 66 HPI (*n* = 69), of which 18 and 20 genes were correlated with leaf rust severity, respectively (Additional Table 6). Hub genes from the ‘R-blue’ module were associated with a reduction in photosynthesis through GO enrichment analysis. From these hub genes only 3 (42 HPI) and 21 (66 HPI) met our criteria for candidate gene selection, with all three genes at 42 HPI and one gene at 66 HPI having a significant correlation with leaf rust severity (Additional Table 6).

### Differential expression analysis of *M. americana* transcripts

**Fig. 5:**
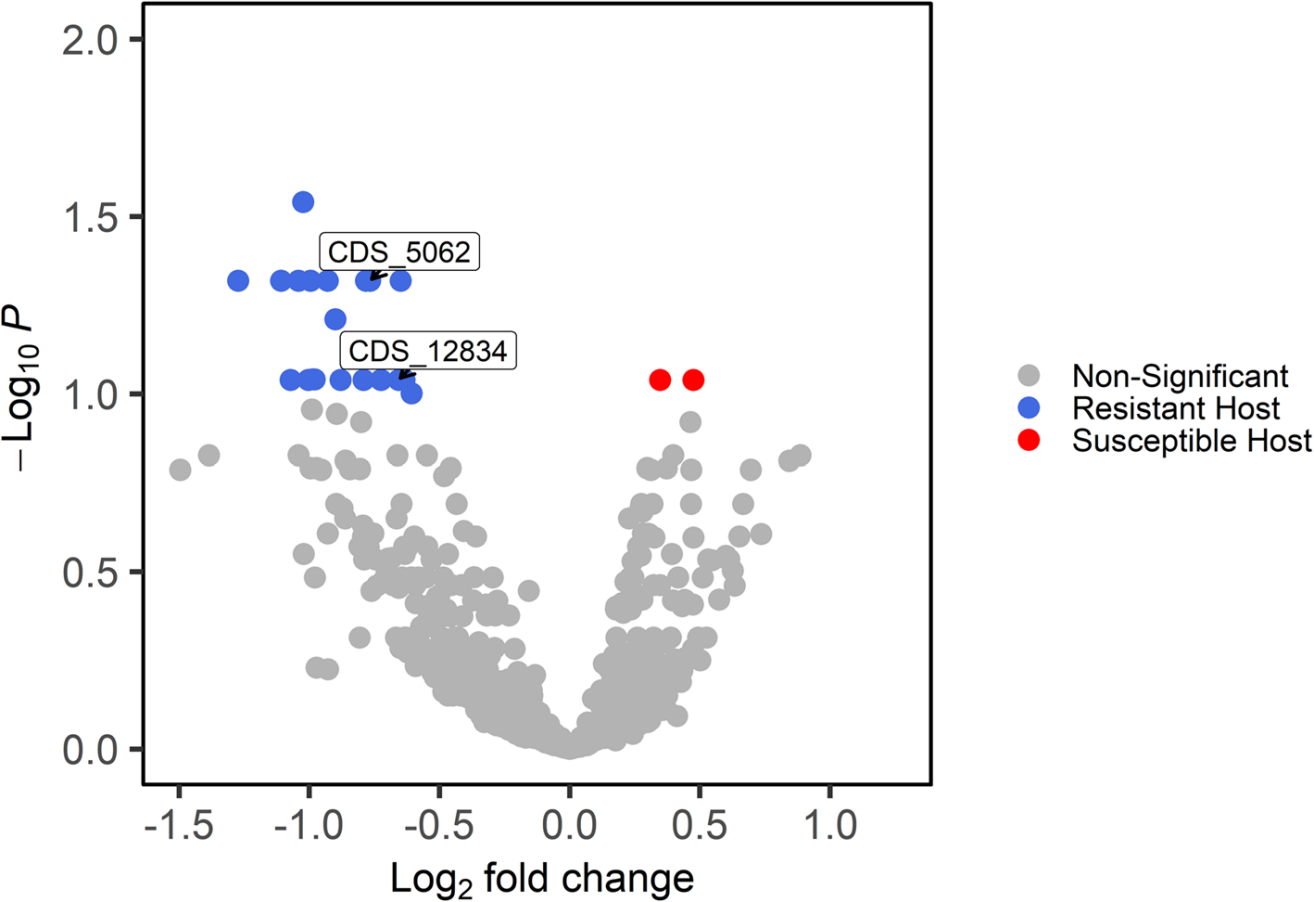
Volcano plot of differentially expressed transcripts of willow rust pathogen *M. americana* at 42 h post inoculation. Positive log_2_ fold change (LFC) indicates up-regulation of *M. americana* genes when grown on the susceptible genotypes (red) while negative LFC indicates up-regulation of *M. americana* genes when grown on the resistant genotypes (blue). Transcripts identified by arrows were predicted to play a role in fungal infection based on in silico effector prediction software Effector P 2.0 (CDS_12834) or through sequence homology to known effectors (CDS_5062). A modified Benjamini-Hochberg adjusted *p*-value cutoff of < 0.1 with no log fold change cutoff was used to determine significance

Total raw reads of the inoculated treatments for each of the 60 willow genotypes (two replicates) were aligned to the *M. americana* reference genome R15–033-03 v1.0 [36]. A direct contrast between genotypes previously identified as resistant and susceptible was performed at each time point (42 HPI and 66 HPI). A total of 22 *M. americana* genes were differentially expressed (FDR = 0.1) between the resistant and susceptible willow genotypes at 42 HPI, yet none at 66 HPI (Fig. 5). The majority of differentially expressed genes were up-regulated in the resistant group (20 genes) as compared to the susceptible (2 genes) (Additional Table 7). A BLAST search of these 22 DEGs was queried against the NCBI nt database [37]. One transcript sequence (CDS_5062) was homologous to a known effector ubiquitin carboxyl extension protein in the plant parasitic nematode *Globodera rostochiensis* [38].

The *in-silico* proteome of the *M. americana* reference genome was analyzed using SignalPv5.0 using default settings to generate an in silico secretome, which resulted in 1779 predicted secreted proteins (Additional Table 8) and analyzed for effector prediction using EffectorPv2.0 using the default settings (Additional Table 9). These proteins were then cross-referenced to the list of differentially expressed fungal transcripts between resistant and susceptible host groups. One (CDS_12834) of the 22 transcripts differentially expressed between the resistant and susceptible hosts was identified as a potential effector.

## Discussion

*Melampsora americana* has previously been shown to be the dominant rust species infecting shrub willow in the northeast United States, yet little is known about the mechanisms of pathogen virulence or host resistance [11, 12]. By applying transcriptomics on willow leaf rust and its host, *S. purpurea*, at 42 and 66 HPI, we defined a coordinated response of DEGs and gene network hub genes associated with a reduction in photosynthesis and an increase in defense response, while simultaneously identifying two candidate effectors for pathogenicity. By leveraging network analysis, differential expression, and eQTL mapping of the host transcriptome, we identified 124 candidate genes associated with a compatible interaction between *M. americana* and *S. purpurea* for future functional characterization.

### Willow transcriptomics

Through the combined use of differential expression, network analysis, and eQTL mapping, this study demonstrated that layering the strengths of each highlights the early response of *S. purpurea* to inoculation by *M. americana* and the varied response between resistant and susceptible genotypes. The contrast between the resistant and susceptible genotypes produced only a moderate number of DEGs. This could be, in part, attributed to the level of resistance observed in the greenhouse compared to the field. While the susceptible genotypes had a high mean severity in the greenhouse with an acceptable CV, the resistant genotypes had a higher mean severity and CV than expected based on the two years of field ratings. This differential interaction is likely based on the environment. In the field there was the potential for multiple rust isolates that relied on the wind to spread, while in the greenhouse there was a single isolate that was brushed directly onto the leaf. A selection of resistant genotypes with a lower, less variable mean severity would have resulted in a greater number of DEGs in this experiment and more refined network and eQTL analyses. Given that the difference in mean severity between our resistant and susceptible genotypes was still significant, the results presented in this study are valuable to research in this pathosystem.

The not-type-specific groups of DEGs identified by contrasting treatments showed many genes in both the resistant and susceptible genotypes that responded similarly to inoculation. A majority of the genes with increased expression in the inoculated treatment at both time points were associated with a defense response. This was supported by the network analysis that revealed that a large number of genes were shared between the ‘R-turquoise’ and ‘S-turquoise’ modules, both enriched for defense response genes. Despite this common defense response, the LFC in expression of hub genes coordinating the resistant response was greater than those of the susceptible response (Fig. 3). By comparing the networks from the inoculated resistant and susceptible genotypes, changes in gene coordination were found that would otherwise be difficult to resolve through a direct contrast given the sample size. Network hub genes are often found to have regulatory control over the other genes in the module, suggesting that small changes in their expression will cascade and resolve in larger changes downstream. This was not unexpected, because prior research has suggested that control of leaf rust severity in the *S. purpurea* F_2_ population used in this study was multi-genic and quantitative in nature [25] and would translate into many genes at lower LFCs that could be difficult to detect. Further evidence for differential coordination is suggested by the resistant and susceptible specific genes that had greater expression in the inoculated treatment. Although both were enriched for defense response genes at 42 HPI, only the resistant specific genes maintained that enrichment by 66 HPI. The susceptible specific genes instead showed enrichment for heat response, aligning well to the ‘S-salmon’ module that split away from ‘R-turquoise’ in the network analysis, but also to the DEGs identified by contrasting the expression within the resistant and susceptible genotypes. Many of the genes up-regulated in the susceptible genotypes at 66 HPI were heat shock proteins, which have been implicated as molecular chaperones that target misfolded proteins for proteolysis and are thought to prevent cell death [39], a benefit to biotrophic pathogens. Not only does this suggest that a potential determining factor in the compatible interaction occurred between 42 and 66 HPI, but it also suggests that genes within the resistant specific group likely play an important role.

A reduction in photosynthesis has been shown in other systems to be an initial response to pathogen attack by redirecting resources toward defense response [40]. Here, co-expression modules enriched for photosynthesis and related terms were negatively correlated with time point following inoculation. While the ‘R-blue’ module was the only one in the resistant network enriched for photosynthesis-related genes, it was split into six separate modules within the susceptible genotypes. In combination with the differential expression results, hub genes of ‘R-blue’ were better able to coordinate resources away from photosynthesis and toward defense response. A faster, more coordinated response in the resistant interaction has similarly been found in the interaction between *Populus* and *M. larici-populina* [41].

Three of the 14 eQTL hotpots detected in this study were enriched for chloroplast components and photosynthesis (C6A at 42 HPI and C16 at 66 HPI) and communication and signaling (C3 at 42 HPI). Although not significantly enriched for GO terms, several defense response genes were associated with all eQTL hotspots. It is likely that the effectiveness of this analysis was influenced by sample size, as power was limited. Despite that, many differentially expressed genes and co-expression module hub genes were connected to an eQTL hotspot, either by direct association or genomic proximity. Based on the intersection of all three analyses, 124 genes predicted to be associated with promoting the defense response and aiding in the coordination of photosynthesis that should be targeted for future studies.

### Rust transcriptomics

As *M. americana* is an obligate biotroph, in silico techniques can narrow down candidate effector genes that are most likely to modulate host immunity. Effector prediction has been a successful initial strategy in the poplar rust pathogen *M. larici-populina* [19, 21, 41, 42] and has led to functional assays that further validate candidate effector function [20, 43]. After in silico effector prediction, Petre et al., 2016 [20] was able to utilize live cell imaging by laser-scanning confocal microscopy in combination with florescent tagged candidate effector chloroplast-targeted protein 1 (CTP1) in *Nicotiana benthamiana* to track cellular localization of the translocated protein. To begin the process of effector discovery and validation of effectors in *M. americana*, we identified two candidate fungal effectors that were differentially expressed between resistant and susceptible hosts. These candidates were discovered based on direct homology to a known effector in nematode (CDS_5062) by using an effector prediction software (CDS_12834). Both transcripts were identified when grown on resistant hosts, possibly indicating that corresponding R-genes exist in the susceptible pool that recognize these transcripts. It was surprising that CDS_5062 showed strong homology to a ubiquitin carboxyl extension effector protein in the nematode *Globadera rostochiensis*, which may be evidence of convergent evolution. In this nematode, it was shown by Chronis et al., 2013 [38] that the peptide is cleaved into a ubiquitin subunit involved in suppression of immunity and a carboxyl extension subunit involved in promoting feeding cell formation. Perhaps the translated CDS_5062 transcripts function similarly, utilizing free ubiquitin as an immunity suppressor. Future proteomic studies will determine if the protein product is similarly cleaved, and functional studies may reveal what role it plays in parasitism.

Both identified candidate effector sequences show promise for future studies, however the overall number of differentially expressed pathogen transcripts identified between the resistant and susceptible groups was quite small. It is possible that this accurately reflects a small number of differentially expressed transcripts and that most of the identified differentially expressed genes play an unknown role in infection. It is also possible that we lacked the proper statistical power to capture the true number of differentially expressed fungal genes and since less than 0.5% of transcripts aligned to the fungal genome, we likely only captured those with the greatest abundance. This could be due to the overrepresentation of willow RNA extracted from the leaf punch samples in the greenhouse experiment resulting in a low number of total genes aligning to the *M. americana* reference genome. This overrepresentation may be due to a deficit of fungal infection structures at these early infection time points, a phenomenon observed in similar rust pathosystems targeting early infection [19, 44, 45]. Additionally, it is possible that the plant RNA extraction kit we used may not have been optimal for extracting fungal transcripts. Future studies utilizing highly-sensitive RNA extraction strategies like laser capture microdissection or haustoria extraction coupled with fungal specific RNA extraction chemistry may achieve greater sensitivity for differential expression studies of *M. americana*. Regardless, in silico prediction of rust effectors remains a challenging task. There are a few species-specific rust effector motifs, but these have not been proven to be suitable for universal predictions across all rust species [46, 47]. As a result, general peptide characteristics such as length, amino acid proportions, and predicted secretion are used as indicators of putative effectors [21, 41, 48, 49].

## Conclusions

This study described the complex changes in the transcriptomes of both the pathogen and host in the *S. purpurea – M. americana* pathosystem using differential expression, network analysis, and eQTL mapping. Differential expression analysis of fungal RNA produced a short list of genes of interest, with two candidate effector genes that were highly expressed when grown on the resistant hosts. Analysis of host gene expression revealed 124 candidate genes that were differentially expressed co-expression module hub genes associated with an eQTL hotspot. Future research could use qRT-PCR to validate differential expression of listed candidate genes produced through this RNA-seq approach. Of particular interest are 14 candidate genes derived from the ‘R-turquoise’ hub genes with significant negative correlations with leaf rust severity and greater expression among the resistant genotypes at 66 HPI. This study represents a step toward developing true understanding of this pathosystem and unlocking the key to breeding shrub willow resistant to this devastating pathogen.

## Methods

### Inoculation of *Salix purpurea* leaves with *Melampsoraamericana* uredospores

Plants were established from dormant stem cuttings in the greenhouse and grown for two months before inoculation. For the inoculated treatment, 1 mg of uredospores of *M. americana* rust isolate R15–033-03 was applied to each of five mature leaves per plant of each genotype using a paintbrush as previously described [12]. Plants were incubated for 12 h in mist chambers at 20 °C with 100% humidity, then returned to greenhouse under 14:10 photoperiod at 24 °C:18 °C respectively. Leaf discs (6.4 mm) were collected using a leaf disc puncher (BioSpec Products, Bartlesville, OK) from two leaves starting with the first fully developed mature leaf on each of three shoots. The inoculated shoots were flagged to help identify inoculated leaves at later time points. To determine the optimal time for tissue collection, two replicated greenhouse inoculation experiments were completed on two ‘Fish Creek’ and two 94006 *S. purpurea* plants (treatments = inoculated and control) and leaf discs were collected every 24 h over the course of 5 d. Based on the analysis of that pilot study data, leaf discs were collected from the full study of 60 genotypes (see below) at 42 and 66 HPI. Each time the leaf discs were collected between 11 am and 2 pm then immediately frozen in liquid nitrogen and stored at − 80 °C until RNA was extracted. Methods described are depicted in Additional Fig. 3. Leaf rust severity ratings of the inoculated treatments were visually assessed based on the percentage leaf area covered in uredospore pustules at 9 DPI for comparison between the greenhouse replication and field survey data. Experimental research and field studies on plants and the original collections were conducted in compliance with all local, state, and federal regulations. Appropriate permissions were obtained for any collections described.

### RNA extraction and 3′ RNA-seq analysis

Frozen leaf disc tissue was disrupted using GenoGrinder 2000 (SPEX CertiPrep, Metuchen, NJ) and RNA was isolated using Spectrum Plant Total RNA Kit (Sigma-Aldrich, St. Louis, MO). Resulting RNA was quantified using a Qubit 2.0 Fluorometer (Thermo Fisher Scientific, Waltham, MA) and quality was assessed using an Experion (Bio-Rad, Hercules, CA). Libraries for 3′ RNA-seq were constructed by the Cornell Institute for Biotechnology (Ithaca, NY) using the Lexogen QuantSeq 3′ mRNA-seq Library Prep Kit (Greenland, NH) and sequencing was completed using Illumina (San Diego, CA) NextSeq500 (1 × 75 bp) technology. Sequencing reads were checked for quality using FastQC Version 0.11.8 [50] and trimmed using Trimmomatic [51] to remove the polyA tail. The RNA-seq data from host genotype 10X-317-029 collected at 42 HPI in Rep 1 was overrepresented as compared to the other samples sequenced on the same lane. Resulting reads from this sample were randomly subsampled to match the mean read depth of all sequenced samples to 125,000 total reads. Trimmed raw reads were aligned to the *S. purpurea* 94006 v5.1 reference genome (6) using the STAR aligner v2.7.5a [52]. Read counts were generated using HTSeq v0.11.1 [53] and differential expression was determined using the R package DEseq2 [54]. Total number of differentially expressed genes was calculated using a direct contrast of the inoculated and control shrub-X-replicate-X-time.

### Selection of F_2_ genotypes for eQTL mapping

This study relied on a *S. purpurea* F_2_ population previously reported in Carlson et al., 2019 [25] that was generated by crossing female clone 94006 and male clone 94001. Two F_1_ individuals from that cross ‘Fish Creek’ and ‘Wolcott’, selected based on growth habit, yield, and resistance to leaf rust, were crossed to generate the F_2_ population. The F_2_ population is comprised of 485 individuals and is planted in randomized complete blocks in Geneva, NY at Cornell AgriTech. The ratings from 2015 and 2017 [25] were used to identify 28 susceptible and 28 resistant F_2_ genotypes by sorting each year by percent severity and identifying genotypes with either consistently high or consistently low severity in both years. Among these 56 genotypes and the two parents and two grandparents of the F_2_ population, the correlation between the 2015 and 2017 surveys was 0.86 with a *p*-value of 8.9e-16. Two plants of each of these 60 genotypes (28 resistant, 28 susceptible, 2 parents and 2 grandparents) were established in 11.4 L pots from dormant stem cuttings planted on June 18 and September 20, 2018 for each of two greenhouse inoculation experiments conducted in separate greenhouse rooms using the inoculation and leaf disc collection procedure described above.

### Differential expression analysis of *S. purpurea* transcripts

Analysis of differential expression was conducted to achieve two aims. First was to identify the differential expression between the resistant and susceptible genotypes through a direct contrast by splitting the samples into six time point by treatment groups (0 HPI-INOC, 42 HPI-INOC, 66 HPI-INOC, etc.). The other was to investigate the differential response to infection by contrasting the control and inoculated treatments within the resistant and susceptible genotypes separately by splitting the samples into six time point by type groups (0 HPI-Resistant, 0 HPI-Susceptible, 42 HPI-Resistant, etc.). After genes with low counts were removed each group was normalized independently in DESeq2 v1.26 [54]. Sample outliers were then identified and removed in R through PCA and hierarchical clustering. Differentially expressed genes were obtained through the ‘DESeq’ function using the designs, gene counts ~ TYPE and gene counts ~ TREATMENT to isolate the contrast of ‘susceptible’ vs ‘resistant’ and ‘inoculated’ vs ‘control’, respectively. Significance was determined based on DESeq2’s adjusted *p*-value, a modified Benjamini-Hochberg false discovery rate, of less than 0.05 and surpassing a log-fold change cutoff of ±1.

To isolate the 42 and 66 HPI inoculated specific DEG in the contrast of ‘susceptible’ vs ‘resistant’, DEG were removed from either 42 or 66 HPI if that same gene was differentially expressed either at 0 HPI or within each time point’s control treatment. Concomitantly, the contrast of treatments, ‘inoculated’ vs ‘control’, results in the identification of type specific and not type specific DEG by first removing genes that were also differentially expressed at 0 HPI and then grouping the remaining genes into resistant, susceptible, or not type specific DEG for 42 HPI and 66 HPI separately. For the purposes of clarity, ‘not type specific DEG’ refers to differentially expressed genes that were detected in both the ‘resistant’ and ‘susceptible’ genotypes. The resulting gene lists for both contrast groups were divided based on the direction of their LFC. Each contrast group was subjected to GO analysis in agriGO v2.0 [55] using a custom background. As the available *Salix* background on agriGO is based on the *S. purpurea* v1.0 reference genome rather than the current v5.1, a customized reference was created that utilized the *Arabidopsis* homologs included in the v5.1 reference annotation file to translate the *Salix* gene ids into *Arabidopsis* gene ids. Significant terms were determined using an FDR of 0.05.

### Network analysis of *S. purpurea* transcripts

Network analysis is used to identify groups of genes that co-express and are often involved in similar biological processes [56]. To focus on the transcriptome-scale differences in response to infection, network analysis was only performed on the inoculated treatment. Samples from all time points from the inoculated treatment were then divided based on type, susceptible or resistant. After counts were filtered and normalized in DESeq2 and outlier samples were identified using PCA and hierarchical clustering and removed, network analysis was performed using a weighted gene co-expression network analysis (WGCNA) in the R package WGCNA [57]. The function ‘blockwiseModules’ was used with the following parameters for both networks; ‘power’ = 12, ‘networkType’ = ‘signed’, ‘minmodsize’ = 20, ‘deepsplit’ = 3, and ‘mergecutheight’ = 0.25. Each module was analyzed for enriched GO terms using agriGO v2.0 [55] as described above in the differential expression of *S. purpurea* transcripts section.

A hypergeometric test using the susceptible network modules as the background was used to compare gene placement across the two networks using a *p*-value of 0.05. Modules found to be enriched for defense related terms or showed a significant relationship with time point were targeted for hub gene analysis. Selected modules were loaded into Cytoscape [34] and analyzed using the plug-in cytoHubba [35]. Module hub genes were identified based on the overlap of greater than 0.8 module membership and greater than 1.5 standard deviations above the mean of log transformed maximum clique centrality (MCC) from cytoHubba.

### eQTL mapping of *S. purpurea* transcripts

Similar methods to Carlson et al., 2019 [25] were used to identify SNPs within the *S. purpurea* F_2_ population for eQTL analysis. Briefly, the TASSEL v5 GBS Discovery Pipeline was used on the full 485 individual population for the initial variant discovery and quality filtering [58]. Reads were aligned to a modified *S. purpurea * 94006 v5.1 reference genome (6), [DOE-JGI, http://phytozome.jgi.doe.gov/] with the 15Z chromosome removed using the Burrows-Wheeler algorithm (BWA) [59]. The resulting 191,650 SNPs were filtered for minor allele frequency greater than 0.01 and 80% missing tolerance before input into LinkImputeR [60]. Setting SNP calls with a depth less than 5 to missing, LinkImputeR’s estimated imputation accuracy of 97.5% was selected, resulting in 47,221 imputed SNPs. Deriving consensus genotypes from multiple sequencing runs of the parents enabled classification of marker types as female or male backcross and intercross markers. Expected segregation ratios based on marker types were tested using a Chi-square test and a Bonferroni corrected *p*-value of 1.7e-6 resulted in 22,570 SNP markers. The 56 selected F_2_ individuals were then isolated and filtered for minor allele frequency > 0.05, with a final marker count of 22,068. Final markers were coded as 0, 1, 2 based on the occurrence of the minor allele.

Sample expression count data were divided into six groups based on the sample’s time point and treatment (0 HPI-INOC, 0 HPI-CTRL, 42 HPI-INOC, 42 HPI-CTRL, 66 HPI-INOC, and 66 HPI-CTRL). Genes with raw counts < 10 across all samples were removed from the analysis then each group was normalized separately in DESeq2 [54] using the ‘estimateSizeFactors’ function and log transformed to account for outlier counts. eQTL detection was performed in MatrixEQTL [61] with the cut-off being *cis-* and *trans*- acting eQTL set at 1 Mb, ‘useModel’ set to modelANOVA with no covariates. eQTL significance for both cis and trans eQTL was determine based on a false discovery rate of 0.05 for *cis-* and 0.1 for *trans*- as calculated by MatrixEQTL. The 42 HPI and 66 HPI inoculated specific eQTL were isolated by comparing the lists of significant eQTL, removing those eQTL from 42 HPI and 66 HPI that were present during 0 HPI and those that were detected at 42 HPI or 66 HPI but in the control treatment. eQTL hotspots were determined based on a 1000 iteration permutation analysis where the number of eQTL per gene was fixed and place randomly among the SNPs without replacement [62]. The maximum number of eQTL occurring on a single SNP by chance was saved from each iteration to form a distribution. The distributions for both 42 and 66 HPI showed that 95% of the maximum eQTL per SNP occurring by chance are less than a threshold of 14 eQTL. To better describe the composition of the genes associating with each hotspot, GO analysis was performed using agriGO v2.0 [55] as described above in the differential expression of *S. purpurea* transcripts section.

### Differential expression analysis of *M. americana* transcripts

RNA extractions, sequencing, and data analysis was performed as described above with the following deviations. Trimmed 3′ RNA-seq reads of the inoculated treatment from Rep 1 and Rep 2 were aligned to the *M. americana* reference genome R15–033-03 v1.0 (https://mycocosm.jgi.doe.gov/Melame1/Melame1.home.html) using the STAR aligner V2.7.5a [52]. A simple contrast was performed for each timepoint by combining RNA-seq reads from both replicates of all susceptible genotypes and contrasting that with the combined RNASeq reads from both replicates of all resistant genotypes. In silico effector prediction was determined by generation of a predicted secretome using SignalP V5.0 using default settings [63]. The resulting secretome was analyzed using EffectorP V2.0 [49] for fungal effector prediction, run with default settings. Resulting transcripts were cross referenced to differential expression data.

## Supplementary Information

**Additional file 1: Additional Figure 1**. Bar graph of total number of differentially expressed transcripts between inoculated and control treatments of Fish Creek (blue) and 94006 (orange) at 1-day intervals for 5 days. Dotted lines represent approximated trends of expression over duration of experiment.

**Additional file 2: Additional Figure 2**. Module eigengene correlations with time point as calculated in WGCNA. Time point was coded as 0, 2, 3. The modules from the resistant network are on the left while the susceptible network modules are on the right. Significance was determined at the 0.05 value. Positive correlations become deeper red while negative correlations become blue.

**Additional file 3: Additional Figure 3**. Schematic of greenhouse experiment. Each leaf was paintbrush inoculated with 1 mg uredospores and image of heavily infected leaf was taken 12 days post inoculation after completion of the experiment. Imaged in bottom is Patrick McMullen.

**Additional file 4: Additional Table 1**. Gene ontology results for each group of differentially expressed genes defined though the contrast of susceptible and resistant genotypes.

**Additional file 5: Additional Table 2**. Gene ontology results for each group of differentially expressed genes defined though the contrast of inoculated and the uninoculated control treatments.

**Additional file 6: Additional Table 3**. Gene ontology results for each module from the network analysis of resistant genotypes.

**Additional file 7: Additional Table 4**. Gene ontology results for each module from the network analysis of susceptible genotypes.

**Additional file 8: Additional Table 5**. Gene ontology results for each eQTL hotspot.

**Additional file 9: Additional Table 6.** List of candidate genes identified through differential expression, network analysis, and eQTL mapping.

**Additional file 10: Additional Table 7**. Summary statistics on differentially expressed *M. americana* transcripts.

**Additional file 11: Additional Table 8**. Signal P predicted secretome.

**Additional file 12: Additional Table 9**. Effector P v2.0 predicted protein summary.

## Data Availability

The datasets generated and analyzed during the current study are available in the Sequence Read Archive (https://www.ncbi.nlm.nih.gov/sra), PRJNA731111: Comparative Transcriptomics of Inoculated *Salix purpurea*.

## Abbreviations

DPI: Days post inoculation
HPI: Hours post inoculation
GO: Gene ontology
DEG: Differentially expressed gene
WGCNA: Weight gene co-expression network analysis
LFC: log_2_-fold change
eQTL: Expression quantitative trait loci
