# Supplementary material for: Comparative transcriptomics and eQTL mapping of response to Melampsora americana in selected Salix purpurea F2 progeny
Source: BMC Genomics. 2022 Jan 22;23:71. doi: 10.1186/s12864-021-08254-1 (PMC8783449; doi:10.1186/s12864-021-08254-1)
Supplement: Supplementary file 1 — Additional file 1: Additional Figure 1. Bar graph of total number of differentially expressed transcripts between inoculated and control treatments of Fish Creek (blue) and 94006 (orange) at 1-day intervals for 5 days. Dotted lines represent approximated trends of expression over duration of experiment. [file 12864_2021_8254_MOESM1_ESM.pdf]

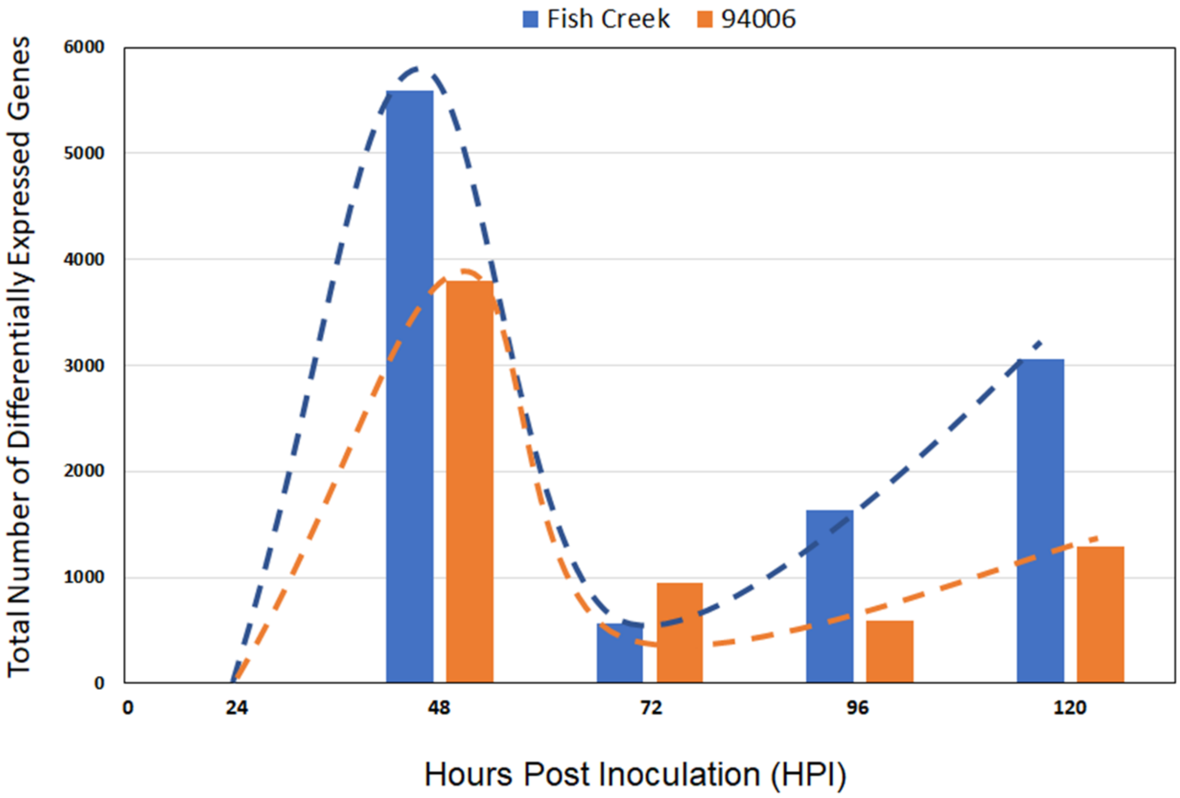

Additional Figure 1: Bar graph of total number of differentially expressed transcripts between inoculated and control treatments of Fish Creek (blue) and 94006 (orange) at 1-day intervals for 5 days. Dotted lines represent approximated trends of expression over duration of experiment.
