# Supplementary material for: Comparative transcriptomics and eQTL mapping of response to Melampsora americana in selected Salix purpurea F2 progeny
Source: BMC Genomics. 2022 Jan 22;23:71. doi: 10.1186/s12864-021-08254-1 (PMC8783449; doi:10.1186/s12864-021-08254-1)
Supplement: Supplementary file 2 — Additional file 2: Additional Figure 2. Module eigengene correlations with time point as calculated in WGCNA. Time point was coded as 0, 2, 3. The modules from the resistant network are on the left while the susceptible network modules are on the right. Significance was determined at the 0.05 value. Positive correlations become deeper red while negative correlations become blue. [file 12864_2021_8254_MOESM2_ESM.pdf]

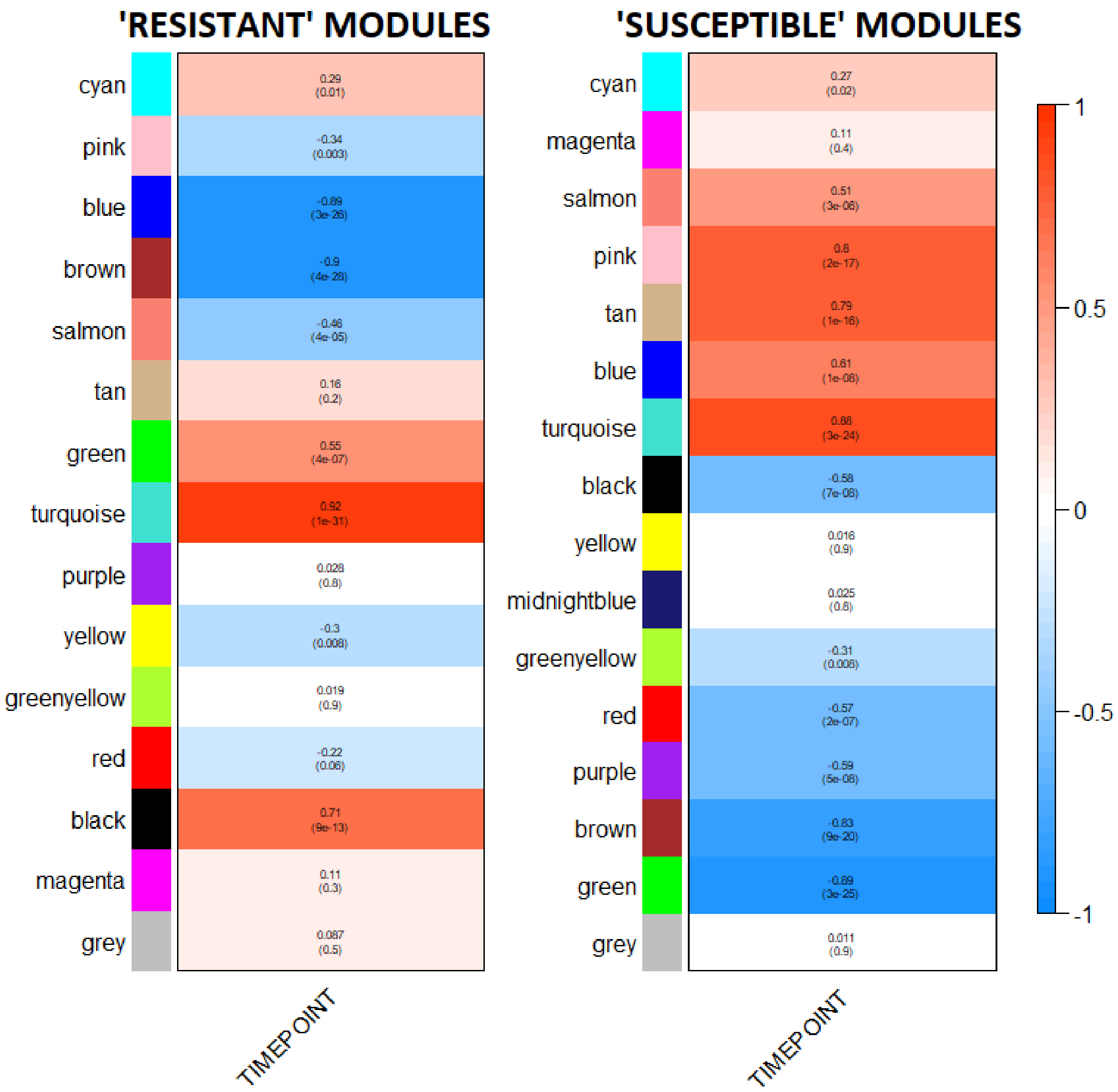

Additional Figure 2: Module eigengene correlations with time point as calculated in WGCNA. Time point was coded as 0, 2, 3. The modules from the resistant network are on the left while the susceptible network modules are on the right. Significance was determined at the 0.05 value. Positive correlations become deeper red while negative correlations become blue.
