# Supplementary material for: Comparative transcriptomics and eQTL mapping of response to Melampsora americana in selected Salix purpurea F2 progeny
Source: BMC Genomics. 2022 Jan 22;23:71. doi: 10.1186/s12864-021-08254-1 (PMC8783449; doi:10.1186/s12864-021-08254-1)
Supplement: Supplementary file 3 — Additional file 3: Additional Figure 3. Schematic of greenhouse experiment. Each leaf was paintbrush inoculated with 1 mg uredospores and image of heavily infected leaf was taken 12 days post inoculation after completion of the experiment. Imaged in bottom is Patrick McMullen. [file 12864_2021_8254_MOESM3_ESM.pdf]

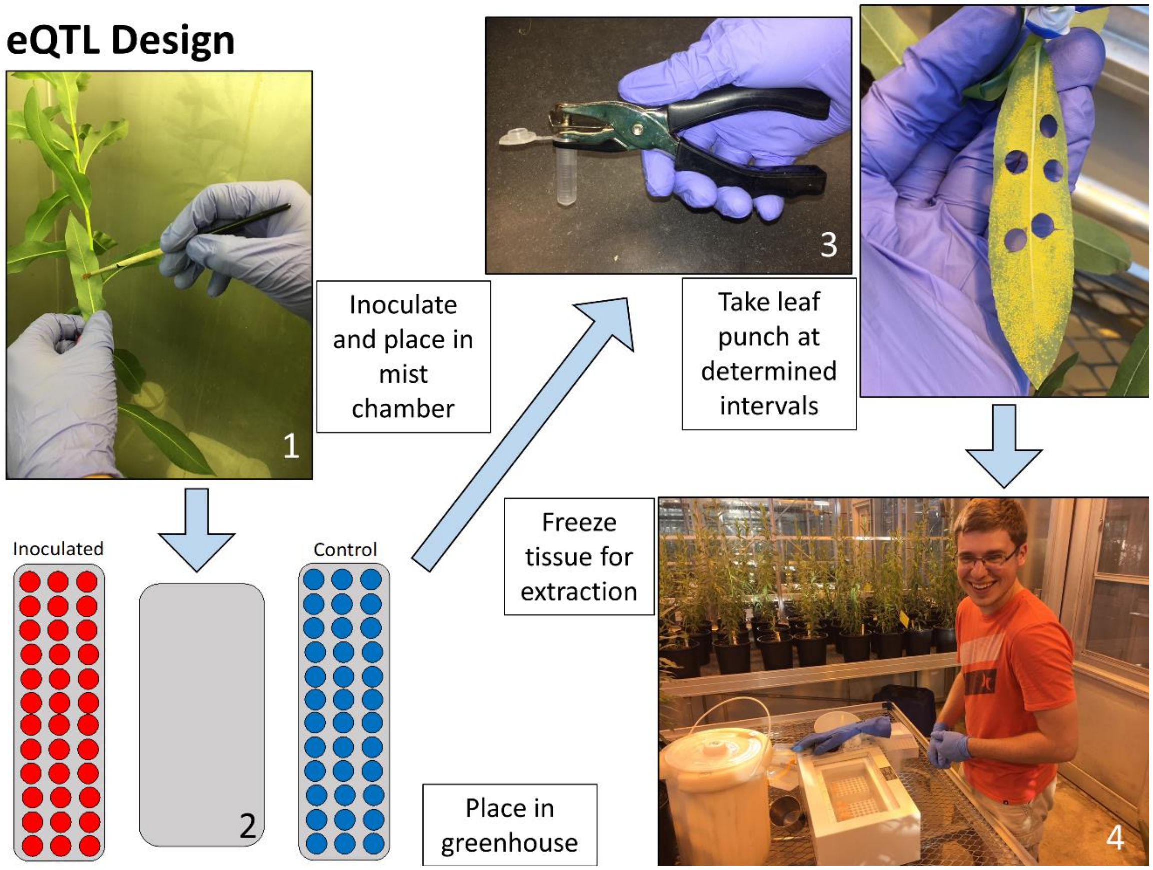

Additional Figure S3: Schematic of greenhouse experiment. Each leaf was paintbrush inoculated with 1mg uredospores and image of heavily infected leaf was taken 12dpi after completion of the experiment. Imaged in bottom is Patrick McMullen.
